# Evolving Scope of Clinical Empathy in the Current Era of Medical Practice

**DOI:** 10.7759/cureus.40041

**Published:** 2023-06-06

**Authors:** Jayakrishnan B, Jothydev Kesavadev, Abhishek Shrivastava, Banshi Saboo, Brij Mohan Makkar

**Affiliations:** 1 Department of Medicine, Educare Institute of Dental Sciences, Malappuram, IND; 2 Department of Diabetes and Endocrinology, Jothydev’s Diabetes and Research Center, Trivandrum, IND; 3 Department of Endocrinology, R&R Hormone Clinic, Jabalpur, IND; 4 Department of Endocrinology, Dia Care, Diabetes Care and Hormone Clinic, Ahmedabad, IND; 5 Department of Endocrinology, Dr. Makkar’s Diabetes & Obesity Centre, New Delhi, IND

**Keywords:** diabetes, communication, doctor-patient, sympathy, empathy

## Abstract

Clinical empathy is one of the most essential tools of medical practice, and it is an act of correctly acknowledging the emotional state of another without experiencing that state oneself. Empathy comprises four components. Mounting evidence exists to support the use of clinical empathy as a tactic for effective health care. Resolving the multi-fold barriers of clinical empathy is important. Clinical empathy is very important in the current era, and a trust-based relationship in patient care is a way to optimal clinical outcomes that can be achieved through better communication and treatment-compliance plans between health care professionals and patients.

## Introduction and background

Empathy has always been and will always be one of the most essential tools of medical practice for health care professionals (HCPs). But it is a common observation that HCPs are facing many challenges in achieving a balance in their relationship with their patients. They even find it difficult to lessen the distance between the patient and themselves to strengthen the connection. The doctors can choose either a narrow technical approach based on their competence or may choose a broadly humanistic approach that is more ambiguous and less reductionist. HCPs' definition of empathy may affect their approach toward patients and their concepts of professionalism [1].

What is empathy

Empathy was initially applied to psychological treatment in the 1950s [2]. According to the centered method, a medical practitioner could momentarily utilize empathy in an effort to learn about a patient's well-being instead of getting to know them personally [3]. According to the Society of General Internal Medicine, empathy is defined as the technique of assessing the emotional state of an individual without experiencing it. According to medical literature, professional empathy can be considered as purely cognitive type that differs from sympathy. 

The Jefferson Scale of Empathy (JSE) originally served to gauge empathy in medical students, and it has evolved into a critical tool for quantitatively evaluating empathy [4]. Physicians and other HCPs who treat patients in clinical settings self-administer the tool for evaluation [2]. The scale comprises 20 questions, and the overall score ranges between 20 and 140 with higher scores signifying better empathic interaction in clinical care.

Difference between empathy and sympathy

The differences between empathy and sympathy are outlined in Table 1.

**Table 1:** Difference between sympathy and empathy Information from references [5-7]

Sympathy	Empathy
Healthcare literature defines sympathy as an emotional reaction of pity towards the misfortune of another, especially those who are perceived as suffering unfairly,	Healthcare literature defines empathy as an ability to understand and accurately acknowledge the feelings of another, leading to an attuned response from the observer.
Sympathy is shared suffering	Empathy is the feeling in which the physician understands the patient’s plight as if the physician were the patient
Sympathy occurs when a physician feels as if he or she is the sufferer	While the doctor empathizes with the patient, he or she also keeps a professional distance from the patient.
Sympathy can be taxing and lead to emotional burnout.	Empathy is employed by skilled doctors to enhance communication and care delivery.

Morse’s components of empathy

Now that we understand the definition of empathy, we shall discuss Morse’s components of empathy. Empathy comprises emotive, moral, cognitive, and behavioral components [8].

· The emotive component is the ability to subjectively share and experience others' psychological state, intrinsic feelings, or emotions.

· The moral component is an intrinsic unselfish force that motivates the practice of empathy.

· The cognitive aspect refers to the therapist's mental capacity to recognize and comprehend another person's emotions and perspectives from a neutral point of view [9].

· The behavioral component is the communicative response to convey the understanding of another person’s perspective.

Cognitive and behavioral components of empathy have received the most attention in clinical practice. However, the importance of a moral stance in the overall philosophy of medicine must not be ignored.

## Review

Benefits of clinical empathy

**Figure 1:**
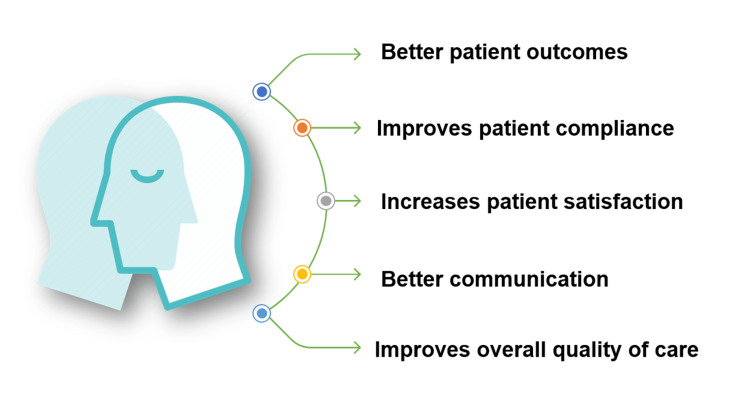
Benefits of clinical empathy Image Credits: Jayakrishnan B (Corresponding Author)

Empathy harnesses optimal patient outcomes through weaving a trust-based relationship between the HCPs and the patients [10]. A physician’s empathetic communication skills have been observed to improve patient adherence and increase patient satisfaction significantly and substantially [11]. Based on patients’ own definitions of quality of care, empathy emerges as a key component in primary care (Figure 1). Medical practitioners who possess a high level of empathy tend to exhibit greater effectiveness in fulfilling their responsibilities and promoting therapeutic improvement [2]. For instance, research on diabetic patients had linked empathy to a successful course of medical care [10,12]. Since patients feel comfortable talking about their concerns and demands, the empathetic professional can comprehend their requirements [2]. Medical professionals' empathy for their patients strengthens their collaboration in creating a treatment plan, hence enhancing the patient's satisfaction with the course of therapy. In this approach, the standard of care is raised, errors are minimized, and a higher proportion of patients benefit from therapy.

Empathy Enhances Non-verbal Communication

Riess and colleagues created a brand-new, simple-to-remember teaching tool that is based on research on the neurobiology of empathy [13]. The acronym EMPATHY can be further expanded as:

E stands for eye contact: It is a key component of social cognition. Eye contact is usually the first signal that one person notices in the other person.

M stands for muscles of facial expression. Human prosocial activity has been demonstrated to be strongly predicted by the capacity to read facial expressions, notably the ability to discern fear.

P stands for posture. Posture is a potent signal of various emotions which can be conveyed independently of facial gestures. The posture of the physician must convey mutual respect and openness. For example, when the physician sits down with the patient at eye level, it conveys that the physician is interested and has time for the patient.

A stands for affect. Consciousness assessment of patients’ affective states is important for improved patient satisfaction, increased treatment adherence, and lower anxiety.

T stands for tone of voice. Tones conveying warmth and anxiety about a patient’s condition have a correlation with no litigation history as opposed to dominant tones.

H stands for hearing the patient carefully. Physicians can hear the “whole patient” by placing the nonverbal signals into the context of patient’s narrative and social world. For this, it is necessary that the physician must not focus exclusively on body parts and physiological functions.

Y stands for your response. While working with difficult patient encounters, a physician’s physiological response is the first signal to proceed with caution.

Empathy Aids in Building a Therapeutic Relationship

It is observed that empathy can aid in building the therapeutic relationship, and several studies have been associated with empathy and the therapeutic relationship to improved psychological and pharmacological outcomes [7]. The core set of common aims and purposes includes initiating supportive, interpersonal communication in order to understand the perceptions and needs of the patient, empowering the patient to learn or cope more effectively with his or her environment, and reduction or resolution of the patient’s problems.

Empathy Promotes Transparency and Confidence and Can Be Directly Effective

Physicians have to deal with tasks that are just as complicated as the things we do in our daily life. Simply relying on logic is not sufficient to determine which issues they should prioritize. Matters that hold emotional significance for the patient are automatically brought to attention of physicians when they are attuned to patient’s emotion. These intuitions should not replace thorough history interviews and other clinical indicators, as they are essential for effective medical practice. Resonance provides expedients but still requires systematic validation [9].

Empathetic doctors not only understand the feelings expressed in their patents’ words but also observe their nonverbal cues. Research suggests that effective communication can alleviate patient anxiety, leading to improved physiological response and better treatment outcomes. When doctors pay attention to their patients’ nonverbal signals, patients tend to feel more at ease and provide more comprehensive medical histories [10]. Research has shown that practicing empathy toward patients can enhance a physician’s sense of fulfillment and purpose in their work. Physicians who adopt an active, psychosocial communication approach are less prone to experiencing burnout than those who don’t. Empathetic connections between patients and doctors can reveal the hidden personal significance behind medical practice, illuminating its true meaning. Physicians who are receptive to being emotionally impacted by their patients can enhance their own experience of practicing medicine [9].

Phases of empathy and key steps to effective empathy

The Three Phases of Empathy

**Figure 2:**
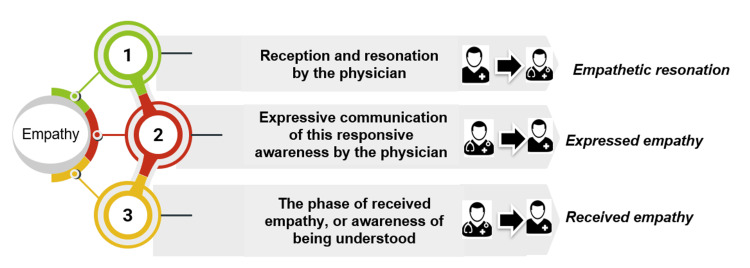
The three phases of empathy Image Credits: Jayakrishnan B (Corresponding Author)

The first phase is empathetic resonation that involves reception and resonation by the physician. The second phase is expressed empathy, that is, expressive communication of this responsive awareness by the physician. The third phase is received empathy that is the phase of awareness of being understood (Figure 2) [14]. Empathy requires physicians to use associative reasoning and emotional attunement to understand the personal significance of their patients' words.

Emotions play a role in shaping our thoughts by creating associations between ideas in an "associative" manner. In addition to logical reasoning, emotions connect ideas that share similarities in sensory, affective, or experiential domains. Associating meaning entails a variety of errors, thus the physician will eventually need to confirm their interpretation with the patient more directly [9]. Creating meaning through associations can be prone to errors, so doctors need to check their understanding with patients directly to avoid misunderstandings.

Key Steps to Effective Empathy

In order to effectively apply a complex concept like empathy, it may be helpful to break it down into its basic components. According to Frederic Platt’s outline [15], essential components of effective empathy are

· Recognition of the presence of intense emotions in the clinical setting such as fear, wrath, anger, and regret.

· The physician should pause and reflect on the patient’s potential emotions. Afterward, the physician can convey the patient’s feelings using their own words to confirm and acknowledge their experience.

· The physician must respect the patient’s coping efforts and offer support and partnership.

Clinical empathy can be improved using the following strategies that address:

· The patient's authority in providing first-person accounts of illness and disability.

· Expanding the concept of empathy to include an action component geared toward relieving patients' suffering.

· The potential value of extending empathy to include the social context of illness [16]. 

Clinical empathy in diabetes care

Diabetes care professionals must provide the best healing environment to patients with diabetes through the art of talking, listening, and caring. The best therapy for patients with diabetes is to listen and empathize with them. Allaying fears and misconceptions of patients may lead to better compliance and adherence. Focus should not be only on medications, but physicians should also help persons with diabetes to understand that the cornerstone of a good outcome lies in the four basic principles: right medication, proper nutrition, daily exercise, and proper lifestyle [17].

Benefits of Clinical Empathy in Diabetics: The Evidence

A study was conducted to test the hypothesis that scores of a validated measure of physician empathy are linked with clinical outcomes for patients with diabetes mellitus. The study included 20,961 patients with type 1 or type 2 diabetes enrolled with one of 242 primary care physicians. The study investigated the relationship between the Jefferson Scale of Empathy scores of participating physicians and the incidence of acute metabolic complications in diabetes patients who were hospitalized in 2009. The study found that patients who were treated by physicians with higher empathy scores had a significantly lower likelihood of experiencing acute metabolic complications such as hyperosmolar state, diabetic ketoacidosis, or coma [1]. The authors concluded that the physician’s empathy is significantly associated with clinical outcome in patients with diabetes mellitus. Also, empathy must be considered as an important component of clinical competence [10]. A separate study by Hojat et al. tested the idea that empathy among practitioners is linked to better clinical outcomes in diabetic patients. The research comprised 891 individuals with diabetes who received treatment from 29 family physicians [12]. The study investigated the correlation between the physicians' empathy scores and the outcomes of their patients. The findings revealed that patients treated by doctors who scored high on empathy had a higher probability of achieving good control of HbA1c, with 56% of patients treated by physicians with high empathy scores achieving good HbA1c control compared to 40% of patients treated by physicians with low empathy scores (P<0.001). The authors came to the conclusion that a key element influencing clinical proficiency and patient satisfaction is a doctor's capacity for empathy [12].

Psychological Factors and Reactions With Negative Outcomes in Persons With Diabetes

Many psychological factors can affect the emotional and psychological well-being of a patient with diabetes. Emotional reactions of patients distressed at the time of diagnosis range from shock, denial, anger, guilt, to anxiety. According to the National Diabetes Service Scheme Australia, diabetes distress can be defined as the emotional burden of living with and managing diabetes. The patients may feel overwhelmed with the demands of self‑management, eventually get frustrated, fatigued, angry, burn out, and in poor mood, due to the difficulty in adapting to the complicated routine, nonadherence to insulin treatment is observed in many patients, which can be associated to psychological barrier linked with fear of needles/injections, insulin initiation, hypoglycemia late complications, and obsessive behavior or overdosing. The psychological disorders associated in patients with diabetes include depression, anxiety, delirium, eating disorder, and schizophrenia. These psychological factors in patients with diabetes can lead to poor glycemic control, lack of self-care behavior and treatment adherence, reduced quality of life, and increased diabetes-related complications [18].

Good Patient-Physician Relationship Promotes Medication Adherence (Patients With Diabetes)

Successful diabetes management requires persons to incorporate complicated medical tasks into their daily routines, makes significant lifestyle changes, and tracks progress and difficulties. A good relationship fosters communication, improves persons’ understanding of illness and treatment, and allows persons to feel comfortable asking questions and participating actively in their own care. The patient-provider relationship also has a demonstrated impact on adherence and health outcomes. Meta-analyses have also found a significant association between physician communication skill and patient adherence and showed that improving the patient-provider relationship has a positive impact on health outcomes across a range of chronic conditions [19].

Barriers to clinical empathy and future perspectives

There are many barriers to empathy. It is argued that listening to patients is made impossible by time constraints. Many doctors still do not consider patients' thoughts and feelings to be a fundamental part of illness and treatment. Discord between clinicians and patients can cause negative emotions. To eliminate these hurdles and clarify the intermediate steps that doctors may employ to practice medicine with true empathy, theoretical and empirical approaches are required [9].

## Conclusions

Clinical empathy has been associated with improved outcomes for patients with diabetes. Apart from pharmacological therapy, patient education, counseling, and psychological support are also important to combat the deleterious effects of diabetes. Aggressive efforts from physicians and motivating persons for compliance are two important aspects in preventing and managing diabetes. Empathy may play a major role in the healing of persons with diabetes.
